# Safety of Xiaflex® (Collagenase Clostridium histolyticum) Treatment for Adult Anterior Urethral Stricture Disease

**DOI:** 10.7759/cureus.45051

**Published:** 2023-09-11

**Authors:** Lucas R Wiegand, Thanh Q Tran, Kevin Heinsimer, Bhavik Shah

**Affiliations:** 1 Department of Urology, University of South Florida, Tampa, USA; 2 Department of Urology, Advanced Urology, Decatur, USA

**Keywords:** intralesional cch, cch, collagenase clostridium histolyticum, urethroplasty, collagenase, xiaflex, anterior urethral stricture, urethral stricture

## Abstract

Male urethral stricture disease is highly prevalent and difficult to treat due to potential complications. Minimally invasive treatments tend to have high recurrence rates, keeping urethroplasty as the gold standard. Collagenase Clostridium histolyticum (CCH) has been used in humans to treat fibrosis in a minimally invasive manner. Herein, we present the preliminary results from treatments of three males with urethral stricture as a feasibility and safety evaluation of the first-in-human CCH treatment for male urethral stricture disease.

## Introduction

Urethral stricture disease can be difficult and expensive to manage. Conservative management involves frequent straight catheterization or indwelling Foley catheter use, which are options that can decrease a patient’s quality of life and increase the risk of urinary tract infections [1]. Minimally invasive treatment options, including direct vision internal urethrotomy, urethral self-dilation, transurethral injection with rapamycin, botulinum toxin, or glucocorticoids, have shown limited efficacy and have fallen out of favor because of poor efficacy and increased morbidity [2,3]. The gold standard for the treatment of anterior urethral strictures is urethroplasty, involving transperineal excision of the stricture with primary anastomosis or substitution of the diseased urethra with grafts. This requires general anesthesia and is often a poor option for patients with significant comorbidities. Additionally, younger, healthier patients are concerned with sexual side effects that can occur unpredictably. Collagenase Clostridium histolyticum (CCH) is an injectable pharmacologic agent that works by hydrolyzing collagen under physiologic environments, with in vitro studies showing CCH specifically selective for collagen types I and III, predominant in Peyronie’s plaques and Dupuytren’s contractures. Urethral strictures, like Dupuytren’s contractures and Peyronie’s disease, are associated with a total increase in types I and III collagen [4]. A recent study investigating injecting urethral strictures with high dose CCH in rats showed a reduction in urethral fibrosis and collagen types I and III expression in the rodents’ urethral strictures [5].

## Case presentation

**Figure 1:**
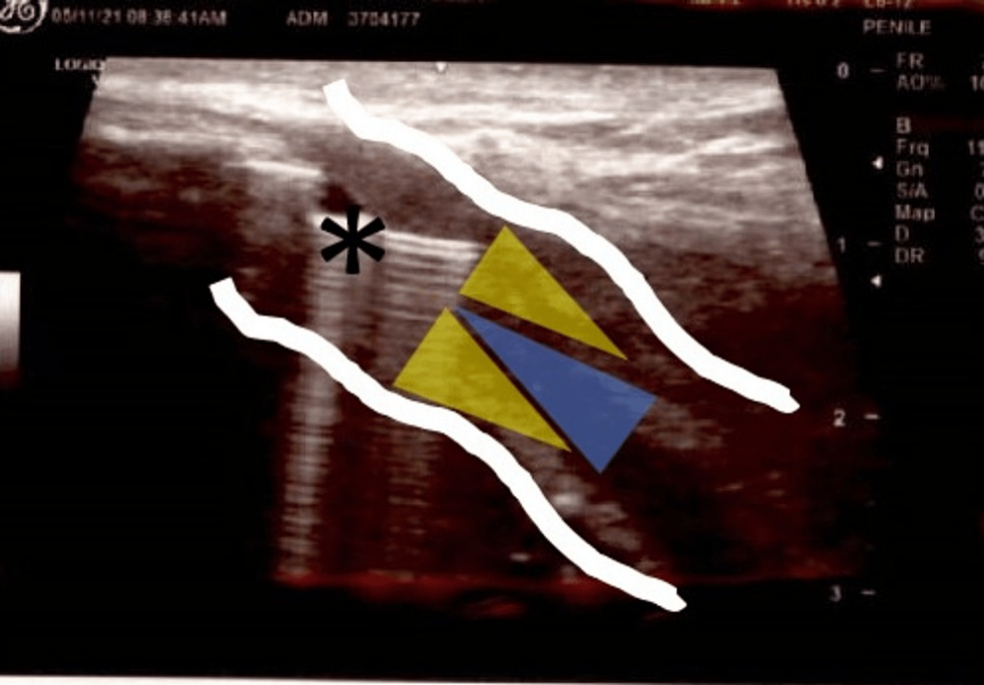
Intraoperative urethral ultrasound Asterisk = cystoscopic injection needle in the dorsal spongy tissue with shadowing. White lines = tunica albuginea. Yellow shaded area = spongiofibrosis. Blue shaded area = urethral lumen.

**Figure 2:**
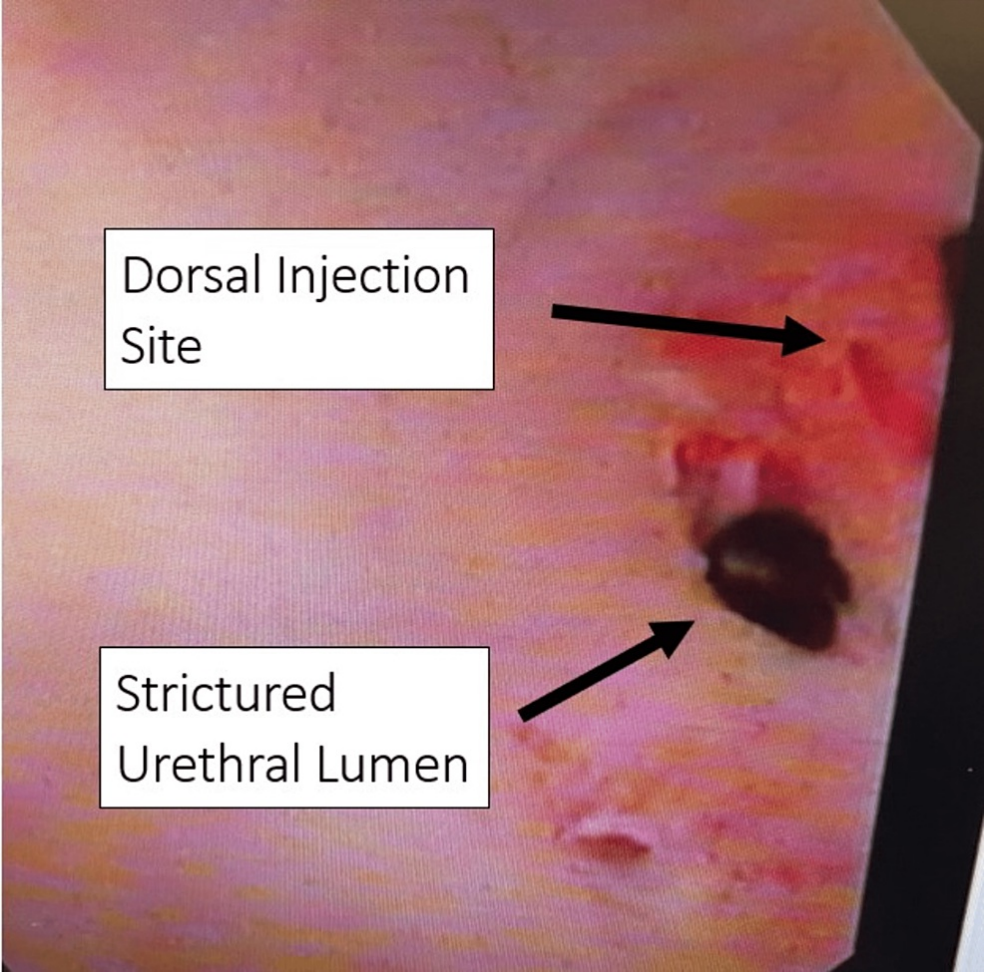
Cystoscopic view post injection

After local institutional review board approval, three white males (age > 18 years) with single bulbar urethral stricture and mild spongiofibrosis underwent one-time treatment with CCH injection. The depth and location of the proposed needle passage were determined pre-procedurally with urethral ultrasonography with the needle in a semi-parallel manner into the plaque to avoid perforation into nearby structures. Injection was performed at a single dorsal site identified on ultrasound as having an appropriate depth of spongy tissue to accommodate the needle without perforating the tunica albuginea (Figure 1). The ventral urethra was avoided, as it is theorized that potential perforation could lead to additional complications, such as fistula formation (Figure 2). Patients underwent instillation of local anesthesia (20 mL of 2% urethral lidocaine jelly), cystoscopy, transurethral injection of 0.08 ml of CCH (0.58 mg of CCH mixed with 0.39 mL of sterile diluent) in the single pre-determined location within urethral stricture, chased by an additional 0.25 mL of reconstituted CCH to allow for clearance of the original 0.08 mL of reconstituted CCH from the transurethral syringe into the urethral stricture/tissue. CCH 0.08 mL was delivered with the 0.25 mL of CCH used to “push” the active volume of CCH through the syringe and into the tissue while ensuring it was not diluted by any other fluid.

Patient 1

Patient 1 was a 78-year-old male (BMI: 25) with a baseline stricture length of 0.69 cm as measured by urethral ultrasound, urethral stricture grade II, and reported American Urological Association Symptom Score/International Prostate Symptom Score (AUASS/IPSS) severe symptoms with “unhappy” quality of life due to urinary symptoms. Baseline measurements are further summarized in Table 1. Following one-time treatment with CCH injection, anticipated short-term (approximately three days duration), mild adverse events reported by the patient included dysuria and hematuria that resolved without treatment. The patient had a cystoscopy performed every four weeks after treatment (for up to day 84), which continued to show mild spongiofibrosis and unchanged urethral stricture. Safety evaluations for up to day 28 post CCH injection are summarized in Table 2. The patient reported lower trending American Urological Association (AUA) scores post-treatment day 14 to day 28 (from severe at baseline to moderate and mild symptoms). AUA score trended up on day 28 for the patient with reported worsening symptoms of urethral stricture requiring dilation. Following dilation, symptoms resolved and AUA scores continued to trend down at day 84 follow-up. At the six-month follow-up, the patient again experienced worsening symptoms of urethral stricture (voiding difficulties) requiring suprapubic catheter placement and subsequently opted for a urethroplasty procedure, which proceeded without difficulty.

**Table 1:** Baseline measurements (n = 3) AUA: American Urological Association; PVR: post-void residual volume.

	Mean ± SD	Patient 1	Patient 2	Patient 3
Age	51 ± 26	78	51	25
Stricture length (cm)	0.9 ± 0.4	0.69	1.47	0.70
PVR (mL)	34.3 ± 27.2	52	48	3
Uroflowmetry – peak flow (mL/s)	7.3 ± 3.2	11	6	5
Spongiofibrosis depth (cm)	0.5 ± 0.1	0.40	0.55	0.70
Spongiofibrosis length (cm)	1.6 ± 0.2	-	1.47	1.73
AUA symptom score	19.3 ± 13.6	21	32	5

**Table 2:** Post CCH injection treatment safety evaluations (n = 3) AUA: American Urological Association; CCH: collagenase Clostridium histolyticum; PVR: post-void residual volume.

Day 2 or day 3:	Patient 1	Patient 2	Patient 3
PVR (mL)	4	52	40
Change in PVR from baseline	-48	+4	+37
Uroflowmetry – peak flow (mL/s)	4	4	4.9
Change in peak flow from baseline	-7	-2	-0.1
AUA symptom score	21	32	4
Change in AUA symptom score from baseline	0	0	-1
Day 14:			
PVR (mL)	27	41	9
Change in PVR from baseline	-25	-7	+6
Uroflowmetry – peak flow (mL/s)	4.8	2	3.7
Change in peak flow from baseline	-6.2	-4	-1.3
AUA symptom score	12	20	5
Change in AUA symptom score from baseline	-9	-12	0
Day 28:			
PVR (mL)	164	74	50
Change in PVR from baseline	+112	+26	+47
Uroflowmetry – peak flow (mL/s)	0	2	4.8
Change in peak flow from baseline	-11	-4	-0.2
AUA symptom score	17	21	5
Change in AUA symptom score from baseline	-4	-11	0

Patient 2

Patient 2 was a 51-year-old male (BMI: 26) with a baseline stricture length of 1.47 cm as determined by urethral ultrasound, urethral stricture grade II, and reported AUASS/IPSS severe symptoms and “mostly dissatisfied” quality of life due to urinary symptoms. Additional baseline measurements are further summarized in Table 1. Following a one-time CCH injection, the patient also reported experiencing anticipated short-term (lasting approximately two days) mild dysuria and hematuria that resolved without treatment. The patient’s post-CCH injection cystoscopies (for up to day 84) continued to show mild spongiofibrosis and unchanged urethral stricture. Safety evaluations for up to day 28 post CCH injection are summarized in Table 2. The patient continued to experience severe AUA symptoms (although scores (20-21) were lower than baseline (32)) post CCH injection and subsequently opted for urethroplasty at approximately 18 months after CCH injection.

Patient 3

Patient 3 was a 25-year-old male (BMI: 29) with a baseline ultrasound-determined stricture length of 0.70 cm, urethral stricture grade II, and reported AUASS/IPSS as per Table 1. The patient reported no adverse reactions following a one-time CCH injection. The patient’s AUA scores remained unchanged up to day 28 follow-up and he opted for a urethroplasty procedure approximately two months after, which proceeded without difficulty. Safety evaluations for up to day 28 post CCH injection are included in Table 2.

## Discussion

Minimally invasive treatment of urethral stricture disease in men with a low complication rate has been a “holy grail” in reconstructive urology. While there was no beneficial effect in patients in this pilot study, initial data did not indicate that CCH injection led to issues with more difficult subsequent urethroplasty, nor was there any evidence of urethrocutaneous fistula formation. Unlike other injections with a less specific mechanism of action [3], CCH mostly affects types I and III collagen, the major component of urethral fibrosis in stricture disease. Other tissues, such as nerves, arteries, and veins, are not affected, which is the underlying reason for the presumed safety of intralesional injection.

Our cases showed that CCH injection was generally well tolerated with low rates of the anticipated adverse events, including minimal hematuria and dysuria (2/3 patients) that resolved without treatment. All patients had a cystoscopy performed every four weeks after treatment (for up to day 84), which continued to show mild spongiofibrosis and unchanged urethral strictures.

Two patients continued to follow up for up to six months post CCH injection treatment. Month six data showed worsening post-void residual volume (PVR). Uroflow showed peak flows that continued to worsen, consistent with the expected evolution of urethral stricture disease. AUA scores continued to trend down from baseline for 2/3 patients, but symptom score severity remained unchanged, likely just reflecting the natural variation of this measurement and our small sample size.

Safe, effective, minimally invasive therapy for male urethral stricture disease is an area of need that merits intense research. Given efficacy data in animal models [5] and our preliminary safety results in humans, further research is warranted to establish whether this may be a viable minimally invasive option for urethral stricture.

## Conclusions

Safe, effective, minimally invasive therapy for male urethral stricture disease is an area of need that merits intense research. Given preliminary safety results, further research is warranted to establish whether this may be a viable minimally invasive option for urethral stricture.

This first-in-human case series shows that CCH is safe at low doses without effect on gold-standard therapy (data may be premature). Future directions will hopefully include increased sample size through multi-institutional participation and adjunctive procedures designed to augment the proposed beneficial effects of CCH therapy.
